# Knowing What We Don’t Know: Model-Based Uncertainty Decomposition for Categorical Sequences

**DOI:** 10.3390/e28070730

**Published:** 2026-06-26

**Authors:** Marc A. Scott, Fulvia Pennoni, Ignacio Bórquez

**Affiliations:** 1 Department of Applied Statistics, Social Science, and Humanities, New York University, New York, NY 10003, USA; 2 Department of Statistics and Quantitative Methods, University of Milano-Bicocca, Via Bicocca degli Arcimboldi 8, 20126 Milan, Italy; fulvia.pennoni@unimib.it; 3 Department of Population Health, Center for Opioid Epidemiology and Policy, Grossman School of Medicine, New York University, New York, NY 10016, USA; ignacio.borquez@nyulangone.org

**Keywords:** entropy, life course analysis, Markov chain models, maximum likelihood estimation, state sequence analysis

## Abstract

State sequence analysis of longitudinal categorical data seeks to synthesize pathways through different dimensions of the life course for descriptive, associative and predictive purposes. Given the number and variety of patterns in such data, measures of the dynamic features of sequences are used to characterize them. One, based on the information-theoretic notion of entropy, measures the uncertainty in the state that will be active at a given time. We customize its use to establish the extent to which we are ignorant, or unsure, of *what happens next* in a dynamic process, conditional on its past. Relying on different Markov chain models for nominal state sequences, we establish multiple measures of uncertainty that allow us to adjust expectations to reflect individual-specific differences and historical information. We establish complementary measures to assess the predictive power of the models in the context of this uncertainty. In so doing, we can summarize and contrast the change in uncertainty associated with different models. As is common in this field, we consider ways in which data can be stratified through demographics and clustering, and how this additional level of partitioning builds a more complete narrative of the social process.

## 1. Introduction

In life course studies, methods for state sequence analysis seek to analyze categorical longitudinal data in a manner that reveals systematic structure through non-parametric or parametric models. Each observation is typically a common-length sequence of nominal states representing important features of the social process, such as co-residence status (e.g., living with one or two parents; living with a partner), education (level and type), labor market participation, potentially tracking industry and occupation of work, health status, and other domains. In fact, the ideas discussed here apply to any process in which successive states are tracked, such as click streams [1], health [2,3,4], and of course genomics, for which many of these methods were initially developed [5].

One goal of sequence analysis is to identify common patterns in sequences either as parameters in a data generating process (DGP), or more broadly as groups or clusters, which themselves may be further examined, characterized, or modeled. Non-parametric approaches to clustering rely on distance measures between observations, metrics for sequence characteristics, and derived sequence structure [6,7,8,9,10]. They are non-parametric because they do not posit an explicit DGP and often rely on algorithms rather than on statistical models. In contrast, parametric models, particularly latent class models, establish groups or delve into the underlying structure governing state transitions, and these include the class of Markov and hidden (latent) Markov models [11] or finite mixture models [12,13,14,15,16,17].

The goals of sequence analysis include identifying (1) the characteristics that describe groups (or predict membership); (2) the dynamics *within* the group over time and whether these dynamics differ between groups; and (3) the potential future (downstream) outcomes associated with each group. In different contexts, Jacobs et al. [18] and Gormley and Murphy [19] examined the first question using a mixture of experts model. The measures presented by Ritschard [8] can be used to describe within-sequence dynamics, while Bartolucci et al. [11], Scott et al. [15], and Murphy et al. [16], among others, do so using statistical models. A mixture regression model [20,21,22] can address the third question.

The gap in the literature is in the second question: how should we evaluate within-group process dynamics? Current best practice might measure some feature of each sequence—a common one is called turbulence [23] and an exhaustive list is given in [8]—and then summarize their distribution. This approach ignores differences that emerge over time, which are central to understanding a dynamic social process. Other approaches examine metrics at the group level, capturing differences over time, but only for aggregated, cross-sectional versions of the data [24,25,26]. We seek methods that characterize individual and population-level dynamics, and we argue that this is best approached by pairing a parametric model with the entropy measure. Entropy is a well-established measure of uncertainty in sequence analysis [27], which in the context of dynamic processes refers to predictability—an essential component of successful modeling and understanding.

We address the following research questions:

- How well do models replicate population-level uncertainty (necessary if we are to substitute models for the empirical data)?
- How much can we improve *individual-level* prediction by knowing the past subsequence?
- How much do explanatory covariates improve predictions?

We wish to model transition dynamics well enough to understand social processes over time (e.g., from education to employment). We will use a parsimonious, interpretable model that can predict different portions of the sequence from its past and covariates, the Markov chain model [11], and will pair it with entropy. We also explore the ways in which pre-stratifying an analysis impacts subsequent modeling and its assessment.

We will evaluate entropy from model-derived predictive distributions in two new and related manners: *locally*, by first conditioning on covariates and past states, and *globally*, as an average for the whole population. We also introduce two parallel measures of accuracy to ensure that the entropy-based assessments reflect a model that predicts meaningfully.

The organization of this paper is as follows. In Section 2, we describe a set of statistical models that are used with categorical sequences. In Section 3, we introduce ways to calculate entropy to evaluate the explanatory power of a model. In Section 4, we illustrate the approach through simulated data and in Section 5 introduce a measure of accuracy to complement the uncertainty evaluation. In Section 6, we apply these evaluations to a study of school to work in Ireland, allowing for stratified or grouped sub-analyses. In Section 7, we indicate guidelines for the practitioner, discuss the connection to more general decompositions of variance, and propose further extensions of the methods. In Appendix A, we provide additional figures and examples to support the main findings.

## 2. Statistical Models for Categorical Sequences

In the analysis of categorical longitudinal data, Markov chain (MC) and hidden Markov models (HMMs) [11] have a predominant role especially when the analysis is focused on transitions. MC models implicitly assume that there are no measurement errors, while HMMs allow for an unobserved state to represent true status that may change dynamically over time. In both forms, many individual time-fixed and time-varying covariates may be available and they can be informative with respect to the transition process as well as the outputs observed at each time point. Further details on both MC models and HMMs can be found in [11].

It is important to note that most sequence analysis techniques use the entire sequence (or a subsequence within) as the unit of analysis. These techniques rely on distance matrices and clustering to organize and synthesize homogeneous subgroups that follow similar patterns or trajectories. These approaches generally do not explicitly account for how transitions between states are experienced or how they correlate with covariates; post hoc analyses explore these concerns separately. MC models can provide insight into this by directly modeling the various transitions between successive states.

There have been attempts to integrate MC models and sequence analysis. Wu [28] noted that standard optimal matching methods used in sequence analysis mathematically “symmetrize” substantively different life course transitions (e.g., from employment to unemployment vs. from unemployment to employment) and do not account for nonlinear temporal dependencies or time-varying covariates. Bison [29] proposed a framework that integrates network and sequence analysis, in which states are conceptualized as successive nodes in a “time sequence network” (a directed graph). Using this approach, typical transitions between states and pathways experienced by the population can be analyzed and modeled. Helske and Helske [30] developed the seqHMM package in R software [31] to model sequences with HMMs and mixture HMMs as an alternative to clustering with latent classes and covariate information. Other recent work in this community reflects an attempt to reintegrate transition models with the life course perspective [11,15,17]. We discuss MC models to introduce the proposed measures of uncertainty and goodness of fit, but the methods transfer equally well to HMMs, and we elaborate on this in the Section 7.

Let us consider response variables $Y_{it}$ with *M* categories labeled from 1 to *M* representing states, i.e., $Y_{it}\in \left\{1,2,\ldots ,M\right\}$, and its corresponding vector of covariates $x_{it}$, considered as fixed (non-random), for i=1,…,n and t=1,…,T. For clarity of exposition we refer to the case of balanced longitudinal (sequence) data, but these models can also be applied when the number of observations $T_{i}$ varies for each sample unit.

### 2.1. Markov Chain Models

MC models describe the transitions from state to state in a probabilistic fashion for data collected over time. An MC model, broadly speaking, is a set of initial probabilities for the first observed state and then a stochastic model for changing state for each newly observed time point. A first-order MC [32] specifies a probability for the state at time *t* given the value of that state at time t−1, so it provides a probabilistic rule for sequence transitions.

The model can be formulated assuming that for t=3,…,T and given $Y_{i,t-1}$, $Y_{it}$ is conditionally independent of $Y_{i1},\ldots ,Y_{i,t-2}$. The model is characterized by initial and transition probabilities that, for every i=1,…,n, are denoted by $\lambda_{i,u}=p\left(Y_{i1}=u\right)$, u=1,…,M, and $\eta_{it,uv}=p\left(Y_{it}=v|Y_{i,t-1}=u\right)$, for t=2,…,T, and u,v=1,…,M, respectively. These parameters are subject to non-negativity constraints, as well as to $\sum_{u=1}^{M}\lambda_{i,u}=1$ and $\sum_{v=1}^{M}\eta_{it,uv}=1$. To enhance parsimony and interpretability, the parameters of the MC model can be subject to various constraints. For instance, imposing time homogeneity implies that the transition probabilities remain constant over time, i.e., they do not depend on *t*. Additional constraints can also be introduced on the transition probabilities to facilitate hypothesis testing [11]. Methods for parsimonious parameterizations of transition matrices of MC models and HMMs can be considered when covariates are available to reduce the number of parameters [33].

In sequence analysis, time-fixed and time-varying covariates are often available. These covariates may be conditioned within the model, allowing it to be time non-homogeneous in a parsimonious manner. The initial probabilities can be parameterized to depend on the vector of covariates in $x_{i1}$, and the transition probabilities are collected in the column vectors $\lambda_{i,\cdot }$ and in the subject and time-specific transition matrices $\Pi_{it}$. For the MC model, the natural parameterization is of multinomial logit type:

(1) $$\log\frac{\lambda_{i,u}}{\lambda_{i,1}}=\alpha_{u}+x_{i1}^{\prime }\beta_{u},\;u=2,\ldots ,M,$$

for the initial probabilities, with the first state taken as the reference. The transition probabilities for individual *i* from state *u* to *v* at time *t*, $\eta_{it,uv}$, are of primary interest, as they comprise $\Pi_{it}={\left\{\eta_{it,uv}\right\}}_{u=1,v=1}^{M,M}$. They are parameterized as follows:

(2) $$\log\frac{\eta_{it,uv}}{\eta_{it,uu}}=\alpha_{uv}+x_{it}^{\prime }\delta_{uv},\;u,v=1,\ldots ,M,\;u\neq v$$

where the logits have the row of the transition matrix as the reference state (i.e., it varies depending on the origin state).

As alluded to above, there are several standard types of MCs, time-homogeneous, time-varying, and conditional on covariates, which can influence the initial and the transition probabilities. The simplest time-homogeneous MC is a “zero-order” chain, or a set of independent draws from a common multinomial distribution, which in the above formulation would set $\alpha_{uv}=\alpha_{u^{\prime }v},\;\forall \;\;u\neq u^{\prime }$, and include no predictors (lack of dependence on current state). This is rarely of interest as it is unlikely to represent a dynamic process well. The first-order, time-homogeneous MC specifies a single set of one-step transition probabilities via estimated parameters $\alpha_{uv}$ without any additional predictors, so the process is static over time, but state dependence is captured. A heterogeneous MC specifies a complete set of one-step transition probabilities for every time point, which is a saturated model and should well-represent processes whose dynamics are changing over time. Setting $x_{it}^{\prime }=I{\left\{t=t^{\prime }\right\}}_{t^{\prime }}$ for all *i*, or indicators for period, and then repeating these for a total of M(M−1) entries allows for $\eta_{it,uv}$ to be dynamically updated for each t∈{1,…,T−1}. This is a highly-parameterized, time-varying, saturated model. If the probabilities from the heterogeneous MC model with M(M−1)(T−1) parameters can be closely approximated via a more parsimonious regression model on *covariates*, then we can learn what factors are associated with the process’s dynamics. If certain subgroups or covariate patterns exhibit very different dynamics that can be modeled, then these conditional models can yield even more precisely estimated pathways.

### 2.2. Maximum Likelihood Inference

Maximum likelihood estimation (MLE) is based on the log-likelihood function, which involves the probability mass function of the observed vectors of response variables $Y_{i}=\left\{y_{i1},\ldots ,y_{iT}\right\}$ for which $l\left(\theta \right)=\sum_{i=1}^{n}\log f\left(y_{i1},\ldots ,y_{iT}\right),$ where θ is the vector of all model parameters. It can be written as two components where the first involves the initial probabilities and the second the transition probabilities considering that

$$f\left(y_{i1},\ldots ,y_{iT}\right)=\lambda_{i,y_{i1}}\prod_{t=2}^{T}\eta_{it,y_{i,t-1}y_{it}}\;,$$

where we have substituted observed states $y_{i,t-1},y_{it}$ for u,v in the probability expression $\eta_{it,uv}$. Therefore, the two components can be maximized separately:

$$l_{1}\left(\theta_{1}\right)=\sum_{i=1}^{n}\log\lambda_{i,y_{i1}}\;,\;\;l_{2}\left(\theta_{2}\right)=\sum_{i=1}^{n}\sum_{t=2}^{T}\log\eta_{it,y_{i,t-1}y_{it}}.$$

The Newton–Raphson algorithm is generally employed to maximize the likelihood functions $l_{1}\left(\theta_{1}\right)$ and $l_{2}\left(\theta_{2}\right)$ separately, on the basis of the corresponding score vectors and the information matrices. We use the LMest package [34,35] of the R software [31] to estimate model parameters. Initialization strategies and convergence criteria for this implementation are illustrated in Appendix A.1.

Once parameter estimates are computed for a given number of states, the corresponding standard errors may be obtained on the basis of different methods. For the MC model, asymptotic standard errors are derived in the usual way through the observed information matrix which is provided by the estimation algorithm. Note as well that the Bayesian Information Criterion (BIC) [36] and other criteria (e.g., Akaike Information Criterion or AIC [37]) can be used to choose the appropriate model complexity. For MC models, the BIC $=-2(l_{1}\left({\hat{\theta }}_{1}\right)+l_{2}\left({\hat{\theta }}_{2}\right))+d\log n$, where *d* is the total number of free parameters in the model and *n* is the number of sequences.

### 2.3. Evaluating Uncertainty Using a Model

Entropy [38] describes the variability in a probability distribution. In the discrete case, with states m=1,…,M, it is the quantity, $H=-\sum_{m=1}^{M}p_{m}\log p_{m}$, where *p* is a discrete probability with countable support M={1,2,…,M}, the set of unique outcome states. Note: use of the natural logarithm is now quite common, but the original Shannon entropy used base 2 [38]. For model-based evaluations [39], we can estimate ${\hat{\omega }}_{tm}$, the conditional probability that the process is in state *m* at time *t*, and this yields time-dependent, conditional entropy

(3) $$H\left(t\right)=-\sum_{m=1}^{M}{\hat{\omega }}_{tm}\log{\hat{\omega }}_{tm},$$

which is one measure of uncertainty over time. However, the conditional probability ${\hat{\omega }}_{tm}$ does not condition on the individual and may be viewed as a population-average approach. We will elaborate on this in Section 3.

### 2.4. Simulated Data DGP

To make the ideas concrete we have a working example, which generates sequences using the following DGP. We simulate data from an MC model with individual time-varying covariates. These may be constructed under different configurations, and we set the number of states to k=3, denoted as A, B and C in the following, with sample size n=1000 and number of time occasions T=31. These states might represent co-residence status, such as with parents (A), alone (B), or with partner (C). For illustrative purposes, we introduce two continuous covariates, each an independent AR(1) process with autoregressive parameter 0.5 and innovation variance equal to 1. For each individual i=1,…,n and time t=2,…,T, the covariates are generated as

(4) $$X_{it}^{\left(j\right)}=0.5X_{i,t-1}^{\left(j\right)}+\varepsilon_{it}^{\left(j\right)},\;\varepsilon_{it}^{\left(j\right)}∼N\left(0,1\right),\;j=1,2,$$

with $X_{i,1}^{\left(j\right)}=\varepsilon_{i1}^{\left(j\right)}$. The covariates are a crucial part of the DGP used to generate the simulated sequences. Initial state probabilities are modeled using a multinomial logistic parameterization as in Equation (1), with $\alpha_{u}=0$ and $\beta_{u}={\left(0.5\;\;1\right)}^{\prime }\;\;\mathrm{for}\;u=2,\ldots ,k.$ For the transition probabilities governed by Equation (2), the intercept parameters are defined as $\;\alpha_{uv}=-\log(\frac{0.9}{0.1}+\nu_{uv}),\;\nu_{uv}∼U\left(0,1\right),\;u\neq v$, and the components of $\delta_{uv}$ are set to values chosen from (0.2,0.3,…,0.7) for $x_{1}$ and (0.8,0.9,…,1.3) for $x_{2}$, making effects stronger for $x_{2}$. Coefficients governing transitions between A, B and C were chosen in a manner that favored transitions to C. These settings also induce a relatively high level of persistence in the MC (common self-loop probability is ∼0.75).

**Figure 1:**
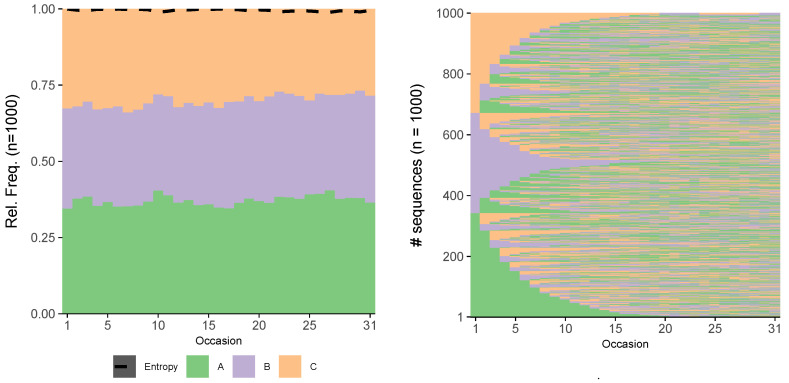
Simulated data: state distribution plot (**left panel**) and index plot (**right panel**). State distribution plots display the proportion of states (A, B, C; see text) at each time point, enabling us to observe the aggregate change in the distribution of states over time. The index plots order subjects by their first observed state and the length of that state and each individual is represented by a single line in the plot. In the left panel, cross-sectional entropy (dashed black line) is nearly constant.

**Figure A1:**
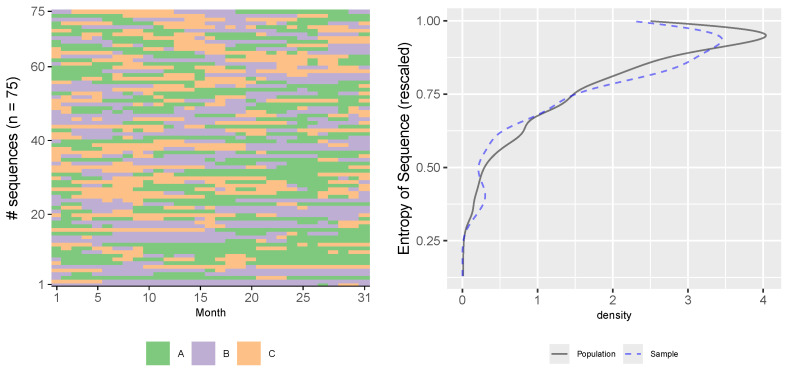
Simulated data: Subsample index plot (**left panel**) sorted by sequence entropy (lowest at bottom). Density of entropy applied to individual sequences ((**right panel**), rotated to refer to (**left panel**)), full population (black, solid) and left panel sample (light blue, dashed).

In Figure 1, we examine these data using exploratory data analysis techniques from sequence analysis, the sequence distribution plot of the cross-sectional, empirical distribution with normalized, cross-sectional entropy overlaid (left panel), and observational-level, color-encoded trajectory plots sorted by initial state, which are known as index plots (right panel) using the package TraMineR [40] in R software [31], a widely used package for sequence analysis in the social sciences. State distribution plots display the proportion of states at each occasion, enabling us to observe the aggregate change in the distribution of states over time, while index plots depict trajectories (and thus state persistence) at the individual level. Even though there is substantial structure in these sequences, their entropy is reasonably large, cross-sectionally, as there are three possible states (A, B, and C) approximately equally likely, but with strong state persistence. If we had only examined entropy cross-sectionally, we would conclude that the process is hard to predict, but this is not the case if we condition (state persistence is high as previously indicated). Moreover, a common alternative, *observation-level* entropy, does not fully capture path-dependent predictability, because it disregards timing. We calculated observation-level entropy, pooling over time, and examined its distribution. The shape suggests high entropy for a majority of observations (consistent with the index plot) and substantial left skew of −1.3. Additional details are given in Appendix A.2 and in particular Figure A1. While the distribution informs us that many sequences change state enough to yield higher entropy, we know very little about the timing of the changes, because this calculation pools time points within individuals (like a “bag of words” model), despite the fact that the process clearly exhibits lagged state dependence. In contrast, our proposed measures will examine population and individual uncertainty evaluations while keeping track of change over time, using a model.

## 3. Entropy Evaluation

### 3.1. Cross-Sectional Entropy

As a reminder, vector $Y_{i}=\left\{y_{i1},\ldots ,y_{iT}\right\}$ is the outcome for subject *i*. Each $y_{it}\in \left\{1,\ldots ,M\right\}$ is a *nominal* state (sometimes called a token), forming a *sequence* of length *T*. As before, let $\omega_{tu}$ be the probability of each state *u* at time *t* derived from an ensemble of sequences, $\left\{Y_{1},\ldots ,Y_{n}\right\}$. In the absence of a model, ${\hat{\omega }}_{tu}=\frac{1}{n}\sum_{i=1}^{n}I\left\{y_{it}=u\right\}$ can be estimated directly. These are cross-sectional frequencies and yield entropy using Equation (3) with a slight change in parameters: $H\left(t\right)=-\sum_{u=1}^{M}{\hat{\omega }}_{tu}\log{\hat{\omega }}_{tu}$.

### 3.2. Model-Based Entropy: Globally and Locally Assessed

As is clear from the index plot (Figure 1 right panel) and the discussion above, states may be highly persistent within person, but cross-sectional (aggregated, between person) entropy suggests otherwise. The advantage to a model-based approach is that entropy can be evaluated individually, conditional on model parameters and covariates *at specific times*, yielding local information that can then be aggregated to a population-level measure. One could argue that one-step transition probabilities can be computed empirically, and thus, uncertainty can be evaluated at any time point conditional on the prior state. In fact, the heterogeneous MC model contains a unique parameter for every period and every transition probability, and the MLEs for the latter are identical to those we construct empirically at each time point. Explicit modeling will allow us to condition on covariates as well, which is potentially more parsimonious and informative than a heterogeneous MC in terms of the social process. As we construct measures of uncertainty over time for individuals, *we need to think carefully about how we condition and aggregate entropy*, because the interpretations differ, as we now demonstrate.

Predictors $x_{it}$ can greatly reduce the uncertainty for any given conditional probability, $\eta_{it,uv}|x_{it},y_{i,t-1}$, which is the probability of a transition from *u* to *v* at time *t* for observation *i*, conditioned on what is known at time *t*. How might one combine these dynamic probabilities to evaluate model-based, cross-sectional variability? We introduce a global and local approach that describe different aspects of model-based uncertainty.

To construct the *global* measure, given *n* observations, we estimate each $\eta_{ir,uv}|x_{ir},y_{i,r-1}$ and then take the expectation of these transition matrices ${\bar{\Pi }}_{r}$, which at time *r* has entries:

$${\left\{{\bar{\Pi }}_{r}\right\}}_{u,v}=\frac{1}{n}\sum_{i=1}^{n}\eta_{ir,uv}|x_{ir},y_{i,r-1}.$$

From this, we can construct the *t*-step transition matrix ${\bar{\Pi }}^{t}=\prod_{r=2}^{t}{\bar{\Pi }}_{r}$, where each term in the product is for sequential times indicated by *r*.

Then the population-level probabilities for each state at time *t* are given by row-vector ${\bar{\omega }}_{t\cdot }=\bar{\lambda }{\bar{\Pi }}^{t}$, where $\bar{\lambda }=\frac{1}{n}\sum_{i=1}^{n}{\hat{\lambda }}_{i,\cdot }$ is a row-vector of averaged initial probabilities estimated for each individual. The *global* measure aggregates first to the population level, forming a single set of transition matrices and initial distribution. If the model represents the process well, this should yield ${\left\{{\bar{\omega }}_{tv}\right\}}_{v=1}^{M}$ that reconstruct the marginal proportions in each state v∈{1,…,M} very closely (they are identical, by construction, in the heterogeneous MC model as implemented in LMest). This leads us to a goodness-of-fit measure: the model-based and empirical marginal state distributions should be similar at each time point. Rather than compare frequencies, as in a Hosmer–Lemeshow test [41], we calculate the entropy of these population-level averages

(5) $$H_{1}\left(t\right)=-\sum_{v=1}^{M}{\bar{\omega }}_{tv}\log{\bar{\omega }}_{tv},$$

which as noted should resemble the empirical entropy of each cross-section, as derived from the state distributions over time. Thus, comparing global entropy to the empirical, cross-sectional entropy over time provides a visual goodness-of-fit test. If the two curves are very different, it suggests a model misspecification and as such is a form of posterior predictive check.

We note that the measures that we introduce are intended to guide model selection. These could be used in conjunction with information criteria such as the AIC [37] and BIC [36], which are provided in the output of the estimation function of the LMest package [34,35]. We expect that the choice of model will not be based on a single criterion, as different features of the underlying process may be of interest to the researcher.

For the *local* version of entropy, for each individual *i*, condition on each $x_{it}$ and past state(s) to construct ${\hat{\omega }}_{it,u(i,t^{-})v}=\eta_{it,u(i,t^{-})v}|x_{it},y_{i,t-1}$, as before, where $u(i,t^{-})$ is the observed state $y_{i,t-1}$ for *i* at time t−1 in the case of a first-order MC. Unlike for global entropy, we maintain the initial value distributions and transition matrices for each observation *i* to compute the entropy of individual, conditional state distributions. We take the average of individual, model-based conditional entropies. This is the proposed formula:

(6) $$H_{2}\left(t\right)=-\frac{1}{n}\sum_{i=1}^{n}\sum_{v=1}^{M}{\hat{\omega }}_{it,u(i,t^{-})v}\log{\hat{\omega }}_{it,u(i,t^{-})v}.$$

If the model is reducing uncertainty at the observation (or unique covariate pattern) level, this quantity should be smaller than the empirical cross-sectional entropy, as well as the global measure $H_{1}\left(t\right)$, because predictions for each individual are assessed before taking the entropy and average. In essence, global measures are the entropy of the population-averaged conditional distributions and local measures are averages of the entropy of conditional distributions.

## 4. Illustration

### 4.1. Simulated Data

Note that when one specifies a model, one conditions on the vector of covariates $x_{ir}$, but these can be a constant (intercept or “1”), yielding a time-homogeneous MC. Alternatively, period indicators would yield a time-heterogeneous MC. These two models are implemented in the lmestMc function of the package LMest. Further details and scripts to replicate these analyses and figures are provided at the URL given at the end of this article. We will refer to these two models as “Homog.” and “Heter.” in the figures that follow. These two models represent the extremes of simplicity and complexity. If transition probabilities are relatively stable over time, the homogeneous MC could represent the process well and is parsimonious. We then estimate the model using the function lmestMc with covariates predicting initial states and transitions to the next state. If covariates predict change in transition rates over time well, and/or capture additional complexity, they could provide a parsimonious alternative to the heterogeneous MC while also providing insight into the mechanisms underlying change over time. Unless otherwise specified, the lmestMc function assumes a number of states in the chain equal to the number of categories of the response variable, and in this simulation, there are 3 states. There are two covariates, used in the multinomial logistic models for both initial and transition probabilities, and we specify the response with a single formula in which “|” separates the initial from the transition models: state~x1+x2|x1+x2. We will indicate this model with the label “Covar.” in the figures that follow. The model specifications are the following:

1. A homogeneous MC model.
2. A heterogeneous MC model.
3. Model with covariates: state~x1+x2|x1+x2.

Model (3) yields a set of multinomial regression coefficient estimates, as in Equations (1) and (2) that allow us to reconstruct a time-heterogeneous transition matrix $\Pi_{it}$ at every time point for each observation *i*. As a reminder, vector ${\bar{\omega }}_{t\cdot }=\bar{\lambda }{\bar{\Pi }}^{t}$ is derived from model-based averages of individual transition probabilities, yielding ${\bar{\omega }}_{tv}$, for v=1,…,M. These are the components needed to compute (global) Equation (5). For (local) Equation (6), we use the individual-level estimates ${\hat{\omega }}_{it,u(i,t^{-})v}=\eta_{it,u(i,t^{-})v}|x_{it},y_{i,t-1}$ (we condition on covariates and prior observed state). These are the probability of a transition from *u* to *v* at time *t* for *i*, and we compute individual entropy at each time point before aggregating. Note that the calculation of time-specific, individual entropy is not possible without some form of model, and this insight is a key contribution.

### 4.2. Model-Based Evaluation of Entropy

**Figure 2:**
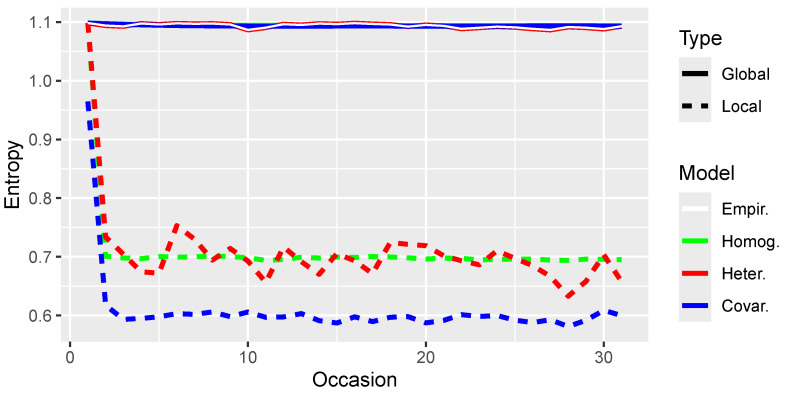
Simulated data: Model-based entropy over time evaluated globally (solid lines) and locally (dashed lines) for three MC models: time-homogeneous, time-heterogeneous, and with individual covariates for initial and transition probabilities. Cross-sectional entropy is shown as a white solid line.

In Figure 2 we display the results of evaluating Equations (5) and (6) over time for the simulated data presented in Section 2.4, for three different model specifications. The conditioned-on-covariate model (3), represented with a blue line, is an attempt to replicate the heterogeneous model’s transition probabilities of the saturated model (in red) in a parsimonious manner.

**Figure A2:**
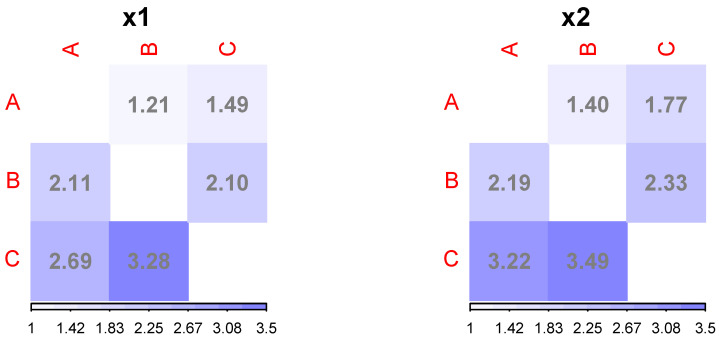
Simulated data: Covariate effects ($x_{1}$, $x_{2}$) presented as odds ratios for the transition probabilities from one of the three states (A, B, and C) indicated in rows of the MC model to another state indicated in columns, relative to staying in the same state.

Referring to the figure, the solid lines reflect global evaluation or population-level entropy, whether based on a model (conditionally) or empirically (from state frequencies) for each time point. For this example, all models are able to reproduce the empirical entropy well. Using the local entropy measure assessed individually before averaging (dashed lines), we find that the homogeneous and time-heterogeneous MC models (green and red dashed lines) reduce the global uncertainty from about 1.1 to a local level of 0.7. At time 1, the uncertainty for these models is maximal, and for those without covariates there is no information about prior state. The model conditioned on covariates has lower initial uncertainty (blue dashed line); the multinomial model, Equation (1), allows us to make better guesses. Quickly after time 1, the model with covariates reduces uncertainty to about 0.6, which is beyond what the heterogeneous chain achieves. Rather than summaries, it is informative to look at local uncertainty over time, which in this case is a fairly smooth function with no apparent spikes. Spikes would signify time periods for which we cannot anticipate the next transition, informing one’s substantive understanding: in addition to whether, we identify *when* we do not know the next categorical state. In Figure A2 in the Appendix A, we visualize the covariate effects governing the transition matrix associated with the covariate model (3) graphically. These are consistent with the fact that predictor $x_{2}$ has a greater impact on transition rates than $x_{1}$.

### 4.3. Entropy Reduction

Global entropy can be based on a range of models, but the time-heterogeneous MC is the natural one to choose if one wishes to document the maximum level of uncertainty in the social process in the absence of covariates (e.g., when will people transition from school to other pathways, such as employment or further education) in a manner analogous to the total variance in a regression problem. In this case, it is a function of time. To the extent that a parsimonious model reproduces this maximal level of uncertainty over time, we can conclude that it fits well in an aggregate sense, similar to how the Hosmer–Lemeshow test [41] assesses the matching of expectations for common blocks (for us, it is over common time points). In contrast, local entropy is assessed at the individual level *before* averaging, so it reflects the information gained by conditioning on a specific individual’s covariates and prior state. It evaluates the ability to guess the next state given all of the information available (rather than population averages).

The extent to which local entropy $H_{2}\left(t\right)$ is substantially lower than the empirical cross-sectional entropy over time is one measure of the predictive power of the model, and this could be framed as the fraction of “explained” variance (which is quantified by entropy). We should not be concerned if the local assessment of a model suggests that it fails to reduce entropy very much or, at the other extreme, it reduces it substantially. Reduction is a function of the number of states, their distribution, and of course, their dependence on covariates or prior states. For example, in the simulated data, local entropy is lowest in the model with covariates—even lower than that of the “saturated” time-heterogeneous model. From a parsimony perspective, this is remarkable, considering that there are 180 (3×2×30) parameters in the “saturated” model for transitions and only 18 (3×2×3) for the parametric model. In this case, the estimated model corresponds to the DGP precisely, with transitions being highly dependent on the included covariates. It may seem surprising that we could not reduce the entropy below 0.6, but even with this DGP’s dependence structure, the probability of leaving the current state is ∼0.25, so the entropy of the two outcomes, (“leave”, “stay”), with probabilities (0.25,0.75), is 0.56, not far from what we achieve in the model with covariates.

## 5. Model-Based Accuracy

As alluded to previously, low uncertainty in an MC model does not guarantee an accurate prediction. A fitted two-state MC model for which the diagonal probability is 0.99 will nearly always predict the same state once initialized and is thus low-entropy. For a true process that alternates between the two states, this is incorrect one-half of the time! Accuracy is how often we make the correct prediction of what actually occurred, but this can be assessed globally or locally as follows. We have defined the average probability at time t∈{1,…,T} of state *v*, which is ${\bar{\omega }}_{tv}$, defined in the paragraph preceding its use in Equation (5). These can be compared to their empirical analogs, ${\tilde{\omega }}_{tv}=\frac{1}{n}\sum_{i=1}^{n}I\left\{y_{it}=v\right\}$, as would be reproduced in a state distribution plot (Figure 1 left panel). This yields the following measure of global accuracy:

$$A_{1}\left(t\right)=1-\frac{1}{2}\sum_{v=1}^{M}\left|{\tilde{\omega }}_{tv}-{\bar{\omega }}_{tv}\right|.$$

The measure is the fraction of probability mass that matches across the two distributions, contrasting observed and expected cross-sectional frequencies for the population as a whole, ranging from 0 to 1. In a perfect match, $A_{1}\left(t\right)=1,\;\forall t$, indicating agreement on the distribution of states. We have correctly matched the uncertainties, but we have not evaluated predictions at the observation level.

We estimate individual-level, conditional probabilities $\eta_{it,uv}$, which may be conditioned on covariates as before. The individual or *local* prediction will be a set of probabilities ${\left\{\omega_{it,u(i,t^{-})v}\right\}}_{v}$, where $u(i,t^{-})$ is the prior state, $y_{i,t-1}$. The accuracy at time *t* can be defined by comparing the conditional distribution to the observed outcome $y_{it}$. For example, if $\omega_{it,u(i,t^{-})v}$ is equal to 0.8 for v=2 and $y_{it}=2$, then we are 80% accurate for that individual at that time point. Assessing predictability at the individual level, the local aspect of this measure is in direct contrast to the global, which operates at the population, distribution level. The local accuracy is the average of individual-level accuracies, now written using indicator functions:

$$A_{2}\left(t\right)=\frac{1}{n}\sum_{i=1}^{n}\sum_{v=1}^{M}I\left\{y_{it}=v\right\}\omega_{it,u(i,t^{-})v}.$$

**Figure 3:**
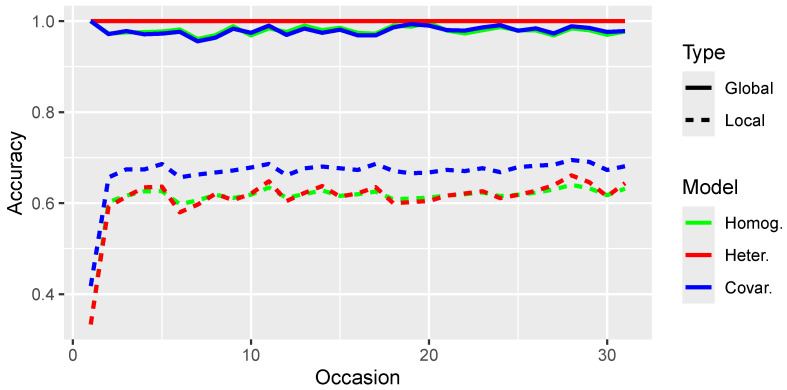
Simulated data: Global (solid lines) and local (dashed) accuracy over time for three MC models: time-homogeneous, time-heterogeneous, and with individual covariates for initial and transition probabilities.

Figure 3 shows the realized values of $A_{1}\left(t\right)$ and $A_{2}\left(t\right)$ for the three MC models. When we aggregate the predictions and evaluate $A_{1}\left(t\right)$, we find that all three models do an adequate job of mirroring the empirical entropy, globally (solid lines near 1). In contrast, the conditional accuracy is lower at the local level, hovering at around 60% for the time-homogeneous and saturated time-heterogeneous MC models after starting at somewhat lower values (the initial state being harder to predict). The model conditioned on simulated covariates $x_{1}$ and $x_{2}$ (blue dashed line; Equation (4)) performs better, with $A_{2}\left(t\right)$ accuracy closer to 70%, an improvement over the saturated time-heterogeneous model. We note that accuracy $A_{2}\left(t\right)$ represents the fraction of correct predictions were they to be drawn at random from the predicted probabilities. We do not threshold the predictions, so whenever there is some uncertainty ($H_{2}\left(t\right)>0$), we will not be 100% accurate. The simulated data’s DGP is conditional on covariates, and these yield observation-period transition matrices from which we predict the next visited state. Upon close inspection, the range of the mean diagonal (typically the largest probability) across all time periods is 71–81%; thus, we can expect to make a poor prediction about 25% of the time. Given this level of inherent uncertainty in the process, we can accept as adequate 70% individual-level accuracy. A key difference between the “saturated” time-heterogeneous model (red) and the model with covariates (blue) is that while the former has 270 (3×3×30) unique probabilities (excepting initial states), at any given time there is a single 3×3 transition matrix available for predictions. In contrast, through conditioning on covariates, a different 3×3 transition matrix may be estimated for every distinct set of covariates at each time, providing more accurate predictions in a parsimonious manner.

Note that fit can be assessed using cross- or relative entropy [42], defined as $H\left(p,q\right)=-\sum_{m=1}^{M}p_{m}\log q_{m}$, where *p* is the “true” distribution and *q* is the one that is to resemble it. For the proposed method, we might take the empirical state distributions over time as the reference, and this would work for a global assessment. However, we make local assessments using individual observations, so these by definition are a point mass at their observed value. Using the above definitions, global cross-entropy is

$$H_{1}^{*}\left(t\right)=-\sum_{v=1}^{M}{\tilde{\omega }}_{tv}\log{\bar{\omega }}_{tv},$$

while local cross-entropy is

$$H_{2}^{*}\left(t\right)=-\frac{1}{n}\sum_{i=1}^{n}\sum_{v=1}^{M}I\left\{y_{it}=v\right\}\log\omega_{it,u(i,t^{-})v}.$$

Given that the term is non-zero for only one *v*, $-H_{2}^{*}\left(t\right)$ is the mean of logged success probabilities, and $e^{-H_{2}^{*}\left(t\right)}$ is the geometric mean version of $A_{2}\left(t\right)$. We restrict the discussion to the more interpretable accuracy measures given that cross-entropy is so similar.

## 6. Application: School-to-Work Transition in Ireland

A study of the school-to-work transition in Ireland was conducted in the 1990s and is described in [43]. Referring to the authors’ initials, the dataset has been named MVAD and is often used as an example when proposing new methods in sequence analysis, as its properties are fairly well understood. The study commences when students are age 16, at which point secondary education is no longer compulsory. Monthly recording of activity ranging from remaining in school (“SC”), further (vocational) education (“FE”), enrolling in higher education (“HE”), apprenticeship training (“TR”), employment (“EM”), or being jobless (not in school; “JL”) define the six distinct states. They are ordered to loosely correspond to common pathways. One can imagine several prototypical pathways, e.g., remaining in school followed by university education or work, further education followed by university or work, or training followed by work, direct employment, and potential for joblessness.

**Figure 4:**
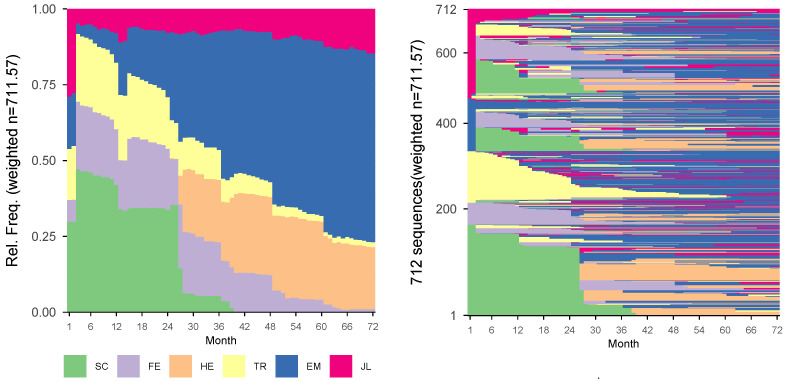
MVAD: state distribution plots (**left panel**) and index plots (**right panel**). The x-axis represents months after secondary education becomes non-compulsory (age 16). Abbreviations: SC = school; FE = further (vocational) education; HE = higher education; TR = apprenticeship training; EM = employment; JL = jobless (not in school).

Referring to the state distribution plot in Figure 4 (left panel), at the start of the study, July 1993 (month 1), individuals may be in five out of the six possible states, and while their distribution is not uniform, the uncertainty as to what the initial state will be is quite large. Once the school year begins (month 3), three choices are quite frequent, training, schooling, and further education, whereas employment and joblessness are infrequent at this stage. Two years later, more than half of those who continued in school enter higher education/university. We can see this in the index plot (right panel), which provides an individual-level perspective: as the schooling spells end, a majority transition to higher education. Similarly, employment tends to follow training spells, and either higher education or employment follow further education. These broad trends may be identified in transition models, but we shall see that path uncertainty remains high at these key junctures.

As mentioned in Section 1, one goal of sequence analysis is to organize observations into meaningful groups, while another is to understand the process within each group. In Section 6.1 we fit MC models to the aggregated MVAD data, establishing the characteristics of the dynamic process for the whole population. In Section 6.2 we discuss stratifying by a demographic, sex, and contrast the groups using the lens of entropy and model-based uncertainty, emphasizing the processes within each group (In the MVAD study, the gender of participants was not measured; only self-reported sex (male or female). When we discuss differences between females and males in our analyses, these likely reflect gender-related social differences.). Then, in Section 6.3, we use non-parametric cluster analysis to divide the trajectories into homogeneous subgroups, such as students on a more academic, university track and others who move directly into the labor market at age 16, before comparing and contrasting these groups with the aid of MC models and an entropy lens.

### 6.1. Aggregated Analysis

We estimate three MC models: one that is time-homogeneous (smoothing the dependence over time); one that is time-heterogeneous (fully capturing the lag-1 dependence); and one in which transitions are conditional on covariates. In the conditional model, we include two covariates that are measured at the start of the study: male; fmpr (whether the father is a ‘professional’). These are time constant. We also include three time-varying covariates set to zero at the start of the study: emp (number of months employed since study began); sch (months in high school since study began); and month (months since study began). Including month as a covariate allows for linear drift in the competing transition probabilities, a parsimonious time heterogeneity for everyone. MLE is carried out as described in Section 2 using the lmestMc function. All models and entropy calculations are weighted with individual sample weights that correct for attrition. In particular, corrections are for any imbalances in type of school attended and destination at study outset (age 16) as well as geographic differences, see [43] and, among others, [44]. The specification for the conditional transition model is state~male+fmpr|month+emp+sch, indicating that initial states are a function of male and fmpr as per Equation (1), while transitions are a function of covariates capturing cumulative state frequencies and time using the multinomial model given in Equation (2). MLE using lmestMc also provides standard errors for the model parameters, allowing for uncertainty assessment and hypothesis testing.

#### 6.1.1. Model Selection

**Figure 5:**
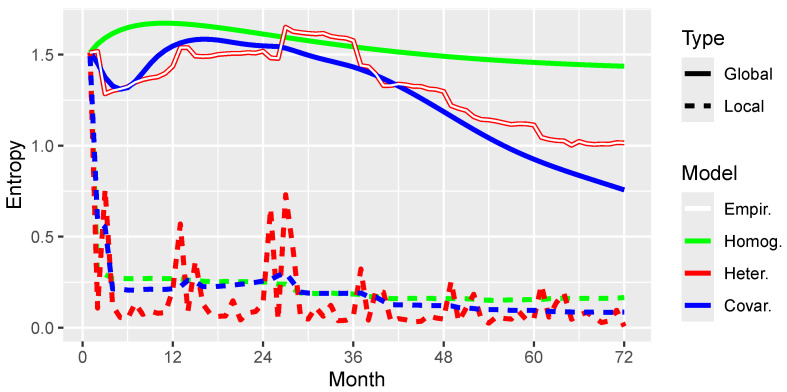
MVAD: Model-based entropy over time evaluated globally (solid lines) and locally (dashed lines) for three MC models: time-homogeneous, time-heterogeneous, and with individual covariates for initial and transition probabilities. Cross-sectional entropy is depicted as a white solid line. The x-axis represents months after secondary education becomes non-compulsory (age 16).

Note that in models like these, information criteria only provide limited guidance. We compare and contrast three model types, with homogeneous and heterogeneous models bounding the set from least to most complex and the model with covariates falling in between. The BICs for these models differ substantially, and comparing the homogeneous MC to the model with covariates, the penalty for extra parameters is worth the improvement in likelihood (2,245,133 vs. 1,982,688). However, the heterogeneous MC model has a much smaller BIC, at 1,540,593. This ordering roughly corresponds to their local entropy as depicted in Figure 5, with the covariate model sandwiched in between the other two.

Each of these models provides a portion of the narrative that is important in describing the social process over time. The homogeneous MC describes the overall rates of change from state to state without adjusting for the timing (a form of smoothing). The heterogeneous MC captures the change over time, but 71 transition matrices (each 6×6) are harder to interpret as a narrative of change (i.e., the model is undersmoothing). It is a saturated model in this context; arguably, its sole function is to establish bounds for predictive uncertainty and accuracy. The purpose of the model with covariates is two-fold: to parsimoniously capture the dynamics of the social process (e.g., school transition) and to identify potentially time-varying characteristics associated with those dynamics. By comparing models, we learn about periods of substantial change from those that smooth less, while we may learn about connections to covariates or overall trends from those that smooth more.

#### 6.1.2. Initial State Model

In this model for the whole population, the regression on initial state estimates that 35% of females with professional fathers will start in school, while 34% of females with non-professional fathers are most likely to start jobless. Males most often start in school (26% for professional fathers vs. 31% for non-prof.), but they have a higher probability of starting in training (17% vs. 25%, prof./non-prof.) than females (7% vs. 12%). These adjust expectations slightly in the first period, but they do not translate to a reduction in entropy, so month 1 local entropy is essentially identical to global entropy.

#### 6.1.3. Transition Model and Uncertainty

In Figure 5, we see that once we pass month 1, a dramatic difference between global and local uncertainty emerges, suggesting that we are able to “explain” much of the uncertainty inherent in MVAD school-to-work pathways conditioning on covariates and past states. This reduction may be attributed to the combination of modeling and maintaining an individual-level perspective when making predictions. There are other aspects of Figure 5 that are noteworthy. First, the saturated time-heterogeneous model (red) with 2130 transition probability parameters has local entropy near zero for much of the interval, with intermittent spikes indicating periods of greater uncertainty. Local entropy close to zero reflects the substantial state persistence of the stochastic process: being in school last month is highly predictive of being in school this month, and so forth for the other states. The spikes suggest something subtle: these are periods of transition, most noticeable in months 24–28, beyond what can be accounted for even with a saturated model. The simpler time-homogeneous model (30 parameters, green) performs better across such transitions in terms of uncertainty but worse overall through a process of averaging or smoothing out such key moments and capturing the transitions common to the process across the full time period. Importantly, the model conditional on covariates (120 parameters) is sandwiched between the other two, which reflects partial averaging or smoothing over the full period combined with using knowledge of past states and covariates. Covariates constructed from cumulative counts of states (e.g., months of employment) are highly predictive of future states, and this is most evident in the last 12 months of the study, for which the model with covariates reduces the uncertainty to very close to zero.

#### 6.1.4. Accuracy

**Figure A3:**
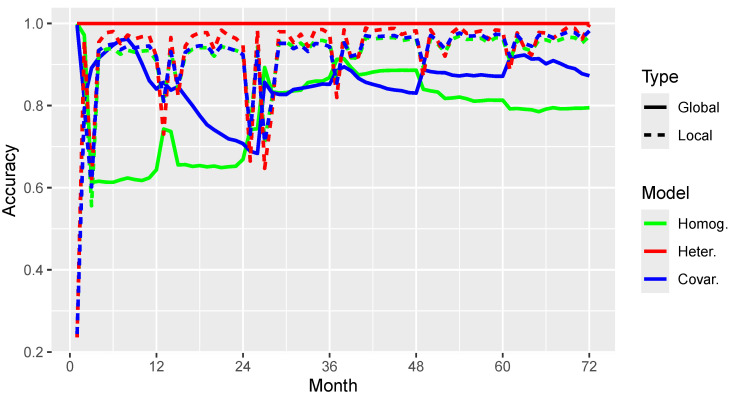
MVAD: Accuracy over time evaluated globally (solid lines) and locally (dashed lines) for three MC models: time-homogeneous, time-heterogeneous, and with individual covariates for initial and transition probabilities. The x-axis represents months after secondary education becomes non-compulsory (age 16).

Accuracy for this model is reasonably high when assessed locally, at near 95% for most models at most times, so we are both certain and accurate (see Figure A3 in the Appendix A). For all models, however, there are spikes of lower accuracy that correspond to the high-entropy spikes evident in the saturated, time-heterogeneous model. Global accuracy requires reproducing the empirical distribution of states at each time point, and this is achieved by the time-heterogeneous model, while the other models have global accuracy closer to 80% most of the time. If we characterize global accuracy as fit in the aggregate, then the model with covariates sacrifices a small amount of fit by smoothing but improves local predictive performance. Accuracy plots help characterize the equivalent of the bias/variance tradeoff inherent in predictive modeling.

#### 6.1.5. Covariate Effects

**Figure A4:**
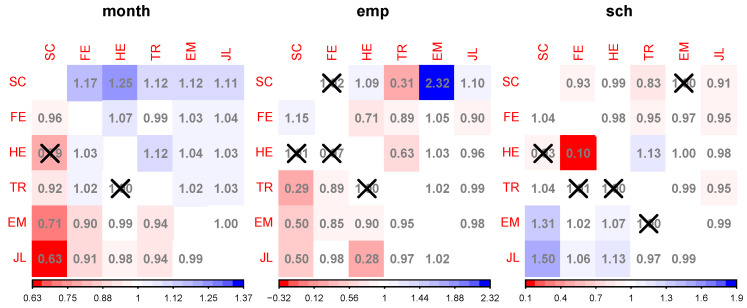
MVAD: covariate effects for months, cumulative employment, and cumulative schooling presented as odds ratios for the transition probabilities from one of the six states of the MC model to another state, relative to staying in the same state. The intensity of cell color (scale centered at OR = 1) indicates the relative magnitude of the estimated coefficient (within matrix). Abbreviations: SC = school; FE = further (vocational) education; HE = higher education; TR = apprenticeship training; EM = employment; JL = jobless (not in school). An X represents an n.s. coefficient.

There are 30 effects on transition probabilities for each of three covariates and an intercept, so we only highlight a few here (see Figure A4 in the Appendix A). As expected, individuals transition less often from employed to school as they age (OR = 0.71; one month difference), from jobless to school (OR = 0.63), and from training to school (OR = 0.92) but more often from higher education to training (OR = 1.12) and from school to higher education (OR = 1.25), all of which are statistically significant (0.05 level). When school is not continued, a lower transition probability likely reflects closing off a pathway back to it. Conversely, those remaining in school open up several pathways, especially to higher education.

The remaining two covariates contain cumulative historical information such as the number of months attending school and the number of months worked once non-compulsory (age 16). These effects may be highly predictive of future transitions because they can characterize an individual as one who stays in school (high cumulative schooling) or one who works a lot (high cumulative employment). However, these effects are estimated simultaneously with months, with which they are correlated. It is best to consider each in combination with the passage of time. For emp there is a large and positive effect (OR = 2.32) on the transition SC→EM (school to employment), which implies that for two groups of students in school, one of which has *in the past* worked one month more than the other, those students have over twice the odds of transitioning to employment. We examined this finding closely and determined that it is partially driven by students who worked during the summer preceding their first non-compulsory school year, which could proxy for class-based differences. A negative effect (OR = 0.10) for sch in the transition HE→FE (higher education to further education) indicates that students currently in college who spent more time in school *in the past* have lower probability of transitioning to further (vocational) education, which is consistent with an understanding of competing pathways. The combined effect of conditioning on the time-constant and historical covariates (cumulative counts) is to reduce entropy from about 1.5 to 0.25, which is over 85%.

#### 6.1.6. State Persistence

**Figure 6:**
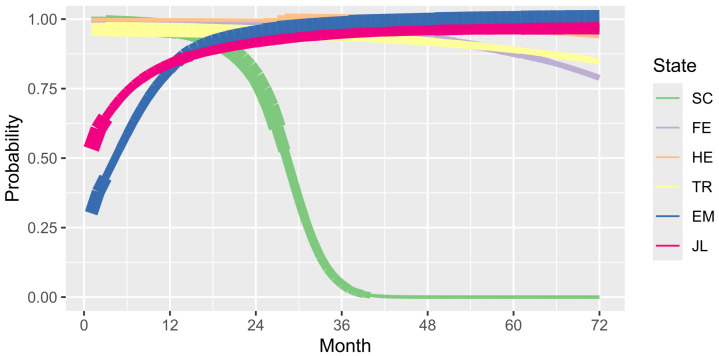
MVAD: state persistence over time for MC model with covariates. Line thickness captures the relative proportion of individuals in that state at that time. The x-axis represents months after secondary education becomes non-compulsory (age 16). Abbreviations: SC = school; FE = further (vocational) education; HE = higher education; TR = apprenticeship training; EM = employment; JL = jobless (not in school).

Estimates of parameters from Equation (2) govern the transitions at each time point. Now, we construct an estimated transition matrix for each time point, basing them on the average of time-specific covariates. For example, at the start of the study, all individuals have 0 prior employment (emp) or school (sch). By the end, it is 72 months later (represented as month), and they have accumulated an average of 27.3 months of employment and 10.7 months of (secondary) school. We use these time-varying profiles as a “mythical average” individual progressing through time (to fix the covariate pattern and focus on the corresponding relative probabilities). In Figure 6, we report the probabilities of the main diagonal of the estimated transition matrices, reflecting state persistence, or stickiness, of each MVAD state as the cohort ages. The thickness of the lines captures the relative proportion of individuals in that state at that time, and the thinnest lines may indicate that no one (or nearly no one) is in that state at that time (prevalence of each state at each time).

We see that for the majority of students, secondary school ends just after month 20, when the probability of continuing a school spell drops precipitously. After this point, joblessness and employment become more persistent, or “sticky” (higher self-loop probabilities), which we interpret as job attachment (or lack of it) becoming more important. These patterns manifest after initial states are achieved, so given female propensity to start in school, persistence implies that females will spend more time in school. Covariate effects on all transitions were previously discussed and are illustrated in Appendix A.3.2. Note that while the state distribution and index plots (Figure 4) are consistent with some of these characterizations, more nuanced statements of likely transitions or differences between groups are made precise by using a model, as we have done, above. To deduce sex-specific state dependence from index plots, e.g., one would have to sort them in a particular manner that manages to reveal it while simultaneously stratifying by sex.

### 6.2. Stratification by Demographics

Groups, or observations that have a common characteristic, such as race/ethnicity or sex, are a natural way to divide data and potentially reduce uncertainty about a process. Stratification can reveal differences in the social process for each group (a form of interaction model). For example, occupational segregation based on sex is common to many labor markets, so knowing the subject’s sex changes the probability of observing a particular occupational status or transition. Social science theory would suggest that women and men have different school-to-work transitions [45]. In the prior model, pooled for men and women, we noted that their initial state distributions varied by sex, which carries forward over time. However, Brzinsky [45] suggests that in many European countries sequence characteristics such as length of spells may vary by sex over time. In Appendix A.4, we stratify the sample by sex, essentially estimating an interaction model and learning more about the mechanisms (and the timing and extent of uncertainty) within each group. By doing this, we may identify sex-specific drivers of variation in their education and employment trajectories.

### 6.3. Cluster-Based Analysis

Groups may be formed using covariates or unsupervised machine learning prior to modeling. Indeed, clustering algorithms and a framework for their use in sequence analysis is well-established [9]. Organizing the data in this manner reduces uncertainty within each cluster, as presumably they reflect some commonality in their content and sequencing. We explore the change in uncertainty induced within clusters now, first by constructing clusters and then by attempting to fit MC models on the smaller groups that are created through clustering. Presumably, these more homogeneous groups formed by clustering can have internal structure that differs substantially between them.

Algorithmic clustering has a long history, and its techniques carry over to sequence analysis quite readily (see Kaufman and Rousseeuw for an overview of cluster analysis [46]). Briefly, these techniques rely on a distance matrix that reflects the dissimilarity of every pair of observations in the sample. There is no natural metric, such as Euclidean distance, for comparing categorical sequences, so the sequence analysis community has developed a wide range of potentially useful distance measures. These sequence dissimilarity measures were summarized and compared by Studer and Ritschard [6]. The tradeoffs for each measure involve the order, timing and duration of states of a sequence; e.g., one may consider two sequences to be very similar if the order of distinct states is the same in them, regardless of the duration of each spell. In this study, we use the longest common subsequence (LCS) distance measure [47], which emphasizes all three characteristics of a sequence. With a distance matrix, one can use divisive or agglomerative algorithms to construct a dendrogram of potential clusterings (the top node is the single cluster of all units, while the leaf nodes are “singleton” clusters, one for each unique sequence). We use the agnes function in the cluster package [48] along with Ward’s distance, a common choice for sequence data.

**Figure 7:**
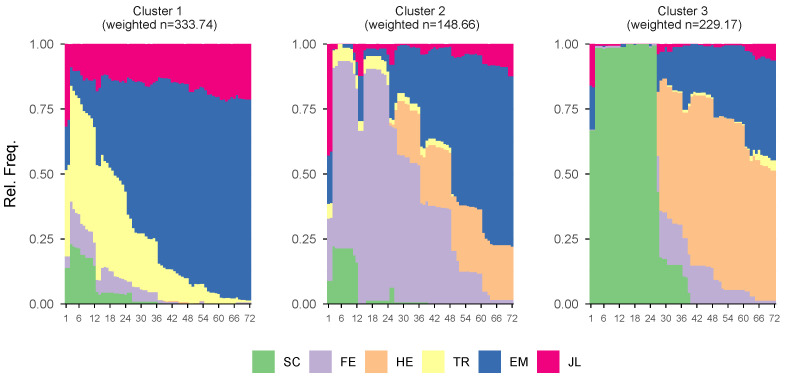
MVAD: state distribution plots for the three-cluster solution. The x-axis represents months after secondary education becomes non-compulsory (age 16). Abbreviations: SC = school; FE = further (vocational) education; HE = higher education; TR = apprenticeship training; EM = employment; JL = jobless (not in school).

The next choice is the number of clusters that balances homogeneity within the groups and heterogeneity between them. The distance matrix is used to assess this between versus within variation in a manner very similar to between and within sums of squares in ANOVA. In fact, analogs to fit measures such as Wilks’ Lambda in MANOVA may be used to choose the number of groups that strike a balance. In sequence analysis, one often uses average silhouette width (ASW) [49] as a measure of fit when choosing clusters. We find that while a four- or five-cluster solution is preferable in terms of ASW, those clusterings reduced the state space of some clusters. While it is possible to build models with different state spaces, models with common states are easier to compare. Thus, we reduced the number of clusters to three for illustrative purposes. State distribution plots for a three cluster solution are given in Figure 7, with cluster 1 reflecting the pathway from training to work, with periods of unemployment; cluster 2 reflecting a further education choice (vocational) that leads to employment or higher education; and cluster 3 primarily reflecting the (secondary) school-to-higher-education (university) path.

**Figure 8:**
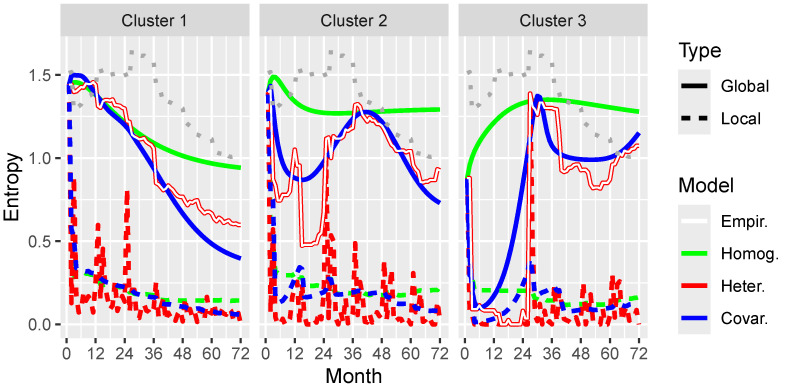
MVAD: Model-based entropy over time evaluated globally (solid lines) and locally (dashed lines) among three clusters for different MC models: time-homogeneous, time-heterogeneous, and with individual covariates for initial and transition probabilities. Grey dotted line represents the population-level common, empirical cross-sectional entropy. Cluster-specific cross-sectional entropy is depicted as a white solid line. The x-axis represents months after secondary education becomes non-compulsory (age 16).

In Figure 8, comparing the aggregate, empirical entropy (grey dotted line) to that of each cluster (white solid line), we see that global entropy has been reduced by creating groups for the data, although the timing of that reduction varies by cluster. The most dramatic difference is for cluster 3, for which global entropy is quite low initially (while in school). Conversely, uncertainty becomes unusually low at the end of the study for cluster 1, as these individuals are highly attached to the labor force. The average global entropy of aggregated MVAD under a model with covariates was 1.28, while for the disaggregated three groups, it is 0.93, 1.04 and 0.82. As expected, clustering is a way of organizing the sequences based on holistically-derived similarities between sequences themselves. In Section 7.2 we will consider the tension between uncertainty reduction between versus within clusters.

We can characterize the global uncertainty within clusters as large but steadily declining for cluster 1, medium with an early dip for cluster 2, and low to medium with a tremendous dip in cluster 3. In cluster 1, training to work describes individuals on a clear path to employment, so the global entropy declines. Beginning with further education, cluster 2 is fairly homogeneous at first, but then larger uncertainty unfolds as there are several paths that follow. School to university, cluster 3, is a more extreme version in the sense that school is a period of great certainty, but as that ends, the next phase is much less clear. Viewed with a prediction lens, locally, a great reduction in uncertainty is possible with the model (∼80%), but the presence of spikes suggests that there are multiple periods of transition and change. As we have seen with prior models, the model with covariates is less susceptible to spikes, or short-term uncertainty, but this comes at a price, as this model performs only slightly better than the homogeneous MC, intermittently.

**Figure 9:**
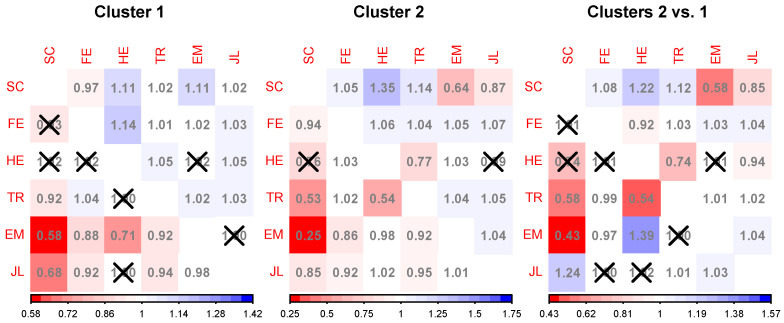
MVAD: Covariate effect for time (month) presented as odds ratios for the transition probabilities relative to staying in the same state for two clusters (panels 1 and 2) and the estimated relative odds ratio comparing cluster 2 to 1 (panel 3). The intensity of cell color (scale centered at OR = 1) indicates the relative magnitude of the estimated coefficient (within matrix). Abbreviations: SC = school; FE = further (vocational) education; HE = higher education; TR = apprenticeship training; EM = employment; JL = jobless (not in school). An X represents an n.s. coefficient.

We describe state persistence for the three clusters in Appendix A.5. Here, we examine the full transition matrix. The graphic was introduced in Appendix A.2 in Figure A2. We return to the multinomial logit model as in Equation (2) and show the estimated odds ratios associated with each transition for a one unit change in the covariate. In Figure 9, we examine the effect of the passage of time by one month. We do this for clusters 1 and 2 and then take their ratio to form the relative odds ratio. We could do this for each pair of clusters, but for illustration we only consider this pair. We also use the estimated standard errors of the parameter estimates to identify non-significant effects (at a 0.05 level; non-significant effects are crossed out).

We see that for cluster 1, as time passes they are less likely to leave employment for nearly any alternative, while the effect is n.s. for joblessness, meaning age is not protective against joblessness. By looking at the distribution plot for cluster 1, Figure 7, left panel, we observe that the proportion of jobless people is more or less constant at each time point, and relative to employment it is non-increasing, consistent with the n.s. finding. In contrast, cluster 2’s risk of changing from employed to jobless is slightly positive and significant (OR = 1.04). Cluster 1 subjects are more likely to move from further to higher education (OR = 1.14) over time, indicating a non-school pathway to higher education (for a small subsample). In this cluster, individuals in school are more likely to move to further education or employment as they age (OR = 1.11 for both). Transition out of training is less differentiated. Cluster 2 consists of substantial further education initially, and nearly all options are more likely with the passage of time (OR = 1.04–1.07) with the exception of school, which of course is rarely, if ever, a return state. Similarly, as subjects age they are less likely to move from higher education to training (OR = 0.77)—it is not a typical pathway. The initial small proportion of cluster 2 in school are more likely to move to higher education (OR = 1.35) and training (OR = 1.14) as each month passes.

Contrasting these two clusters, in the third panel, movement over time from employment to higher education is more indicated for cluster 2 as compared to 1 (OR = 1.39), due in part to the infrequency of higher education in cluster 1. Transitions from school to employment are less common as time progresses for cluster 2 (OR = 0.58) while they are relatively more common to higher education (OR = 1.22). There are other comparisons one could make, but many reflect the structural constraints (e.g., non-return to school) or substantial differences in the proportion in each state across clusters.

## 7. Summary and Discussion

### 7.1. Summary and Guide

In this article, we define two forms of model-based assessments of entropy and accuracy for sequence data. Global assessment tracks predicted marginal distributions of states, which, compared to the empirical analog, allows for a visual inspection of fit. Local assessment includes all of the historical information utilized by a model to make individual predictions of subsequent states. This quantifies the reduction in entropy/uncertainty as we move from population averages to individual predictions, via a model. A model-based approach to entropy assessment moves beyond static, cross-sectional assessments and pooled individual ones. While graphical displays and clustering techniques can reveal some of the same patterns, models with covariates allow us to quantify when, by how much, for whom, and under what conditions we are able to predict portions of the pathway based on its past, informing one’s understanding of the social process. We have demonstrated the utility of these measures in the context of simulated data and school-to-work data in Ireland (MVAD).

**Figure A12:**
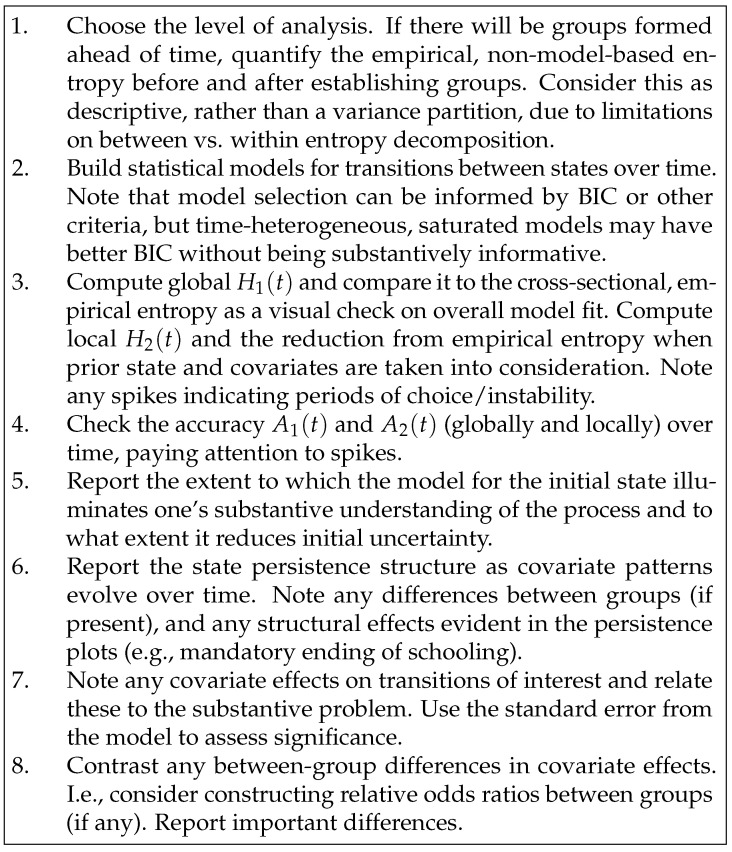
Guidelines.

For the aggregate MVAD analysis, we followed a particular order to assess the models, and to quantify what was known and unknown about the process. An extended set of guidelines for practitioners implementing this model-based entropy approach for sequence data is presented in Appendix A.6, Figure A12. The methodology proposed in this paper was implemented in R Version 4.5.2 [31]; the code for estimating entropy and accuracy and analyzing the data is available at the URL given at the end of this article.

### 7.2. Between vs. Within Uncertainty

Given the importance of groups in sequence analysis, we examined MVAD through this lens, first by stratifying by a time-constant covariate (i.e., sex) and then by clustering. This introduces additional uncertainty *between* the groups, while almost surely reducing the uncertainty *within* them, a form of partitioning quite common with continuous data [50].

While not a direct analog, we can imagine a variance partitioning of sequence data based on the mixture model framework [20]:

(7) $$f\left(Y_{i}\right)=\sum_{k=1}^{K}\pi_{k}f_{k}\left(Y_{i}|\Theta_{k}\right),$$

where $Y_{i}$ is a vector of states in the sequence (as before) for individuals i=1,…,n, while within cluster *k*, $f_{k}\left(Y_{i}|\Theta_{k}\right)$ models the *within* sequence process and $\pi_{k}$ is the weight of the mixture component, with $\sum_{k}\pi_{k}=1$. The $\pi_{k}$ may be modeled further using a multinomial distribution with baseline covariates $x_{i1}$ predicting the component, *k*. Indeed, Roeder et al. [51] and Jones et al. [52] link a conditional assignment to clusters for longitudinal, limited dependent outcomes, but not for the general nominal case, and Helske and Helske [30] implement a mixture regression in which the regression kernel is an HMM.

Equation (7) yields a model-based estimate of the *between*-class entropy, $H^{B}=-\sum_{k=1}^{K}\pi_{k}\log\pi_{k}$, independent of time, since only group membership is uncertain. Within-class entropy conditioned on group membership is $H_{k}^{W}\left(t\right)=-\sum_{m=1}^{M}\omega_{ktm}\log\omega_{ktm}$, where $\omega_{ktm}$ are based on the model $f_{k}$. Arguably, $H^{B}$ and $H_{k}^{W}\left(t\right)$ establish a partition of the uncertainty. However, there is no notion of total uncertainty, as the sum of between- and within-group entropy is not a constant. To understand this, note that as the number of groups or clusters *K* increases, the between-group entropy will tend to increase. Say that group sizes are roughly equal at $\frac{1}{K}$. Then, entropy is a simple function of the number of groups, with $H_{max}^{B}\left(K\right)=\log\left(K\right)$, which is monotonically increasing. As the groups become more homogeneous, the *within-group* entropy should decrease, but the amount is not constrained in any way. This suggests that the partitioning of uncertainty into between and within variation with categorical sequences is more complicated than doing so with its analog, longitudinal continuous outcomes. We focus on the evaluation of *within* sequence uncertainty as it is linked to narratives for the dynamic social process and use existing approaches such as clustering or stratification to establish groups beforehand, if appropriate.

### 7.3. Extensions

Although we introduce the new entropy measures by illustrating them through the discrete-time MC model of the first order, it is worth mentioning that it is sometimes more realistic to consider a latent process underlying the observed sequences [53]. In this case, an HMM may be considered in which the underlying latent process follows a (usually first-order) Markov chain and the response variables are assumed to be conditionally independent given the latent variable (this is also defined as local independence). In this context, the HMM without covariates postulates that the true state is measured with errors. Therefore, the true states correspond to latent (hidden) states. In this way, it is possible to compare the MC models and HMMs, select the best model for the sequence under study, and evaluate the estimated entropy for both models in order to quantify the effect of such errors.

Another flexible class of HMMs includes individual covariates and also the lagged response variable. Generally, the model is specified by incorporating covariates directly into the model responsible for observed outputs. This is done via a suitable parameterization of the conditional distribution of the response variables given the latent process, thus assuming their direct association with the observed response. In this case, the latent variable captures the variation in person-period behaviors that accounts for the effect of unobserved heterogeneity on the response, or the residual variability due to unobservable factors. This model allows for serial dependence by adding the effect of the lagged response variable as an explanatory variable [11]. In this way, the local independence is suitably relaxed and the so-called state-dependence effect can be evaluated, namely, the impact that experiencing a certain situation at a certain time has on the probability of experiencing the same situation in the future, once all the other observable and unobservable explanatory variables have been accounted for [54]. The HMM formulated in this way can be seen as an extension of the dynamic logistic model [55]. In this context, deeper reductions in local entropy are expected compared to the MC model with covariates, although this largely depends on the temporal dynamics of the observed sequences.

Another specification of the HMM is one in which covariates directly influence the latent structure—that is, the initial and transition probabilities of the Markov chain. In this case, individuals are first classified into distinct clusters, defined as subpopulations of subjects exhibiting similar behaviors, for example, those alternating between periods of unemployment and training in the MVAD dataset. The model allows us to analyze the transitions between clusters and also to quantify the effect of observed covariates on both the initial state and the probabilities of moving to different clusters over time. Practical examples of this modeling approach are given in [56,57] for longitudinal and life course data. In this context, the hidden process is time-homogeneous, and therefore it is interesting to compare the expected decrease in entropy with that of the MC model with covariates.

### 7.4. Concluding Remarks

A limitation with the set of models examined in this article is that we are conditioning only on the prior state (one step), but this can be generalized in many ways, e.g., including the HMM [11] or with mixture transition models [58]. Embedding MC models and HMMs within a finite mixture framework has been discussed, and this type of extension could yield further insight into the social process. We note that joint models for within and between processes can shift the interpretation of model parameters and the clusters themselves [19]. We have formulated these measures in a model-based setting. Extending them to non-parametric models may be possible by conditioning on subsequences in a manner analogous to that used in missing data imputation for sequences [59,60].

Global and local measures of uncertainty along with accuracy provide a set of complementary tools to build narratives for state sequence analyses. Global measures take a population perspective that may indicate a lack of fit or specific periods for which a model is misspecified. Local uncertainty reflects precision of predictions that must also be assessed for accuracy. Models with covariates identify factors that yield accurate predictions, and the proposed measures make it precise when these models are more and less effective at that task. These methods serve as a natural complement to existing descriptive, clustering and visualization techniques, and they may be extended to accommodate a variety of model-based approaches. With these tools, we quantify *how much* we know or do not know (the uncertainty of a future state), and we identify *when*, *for whom*, and *under what conditions* this may be problematic. 

## Figures and Tables

**Figure A5:**
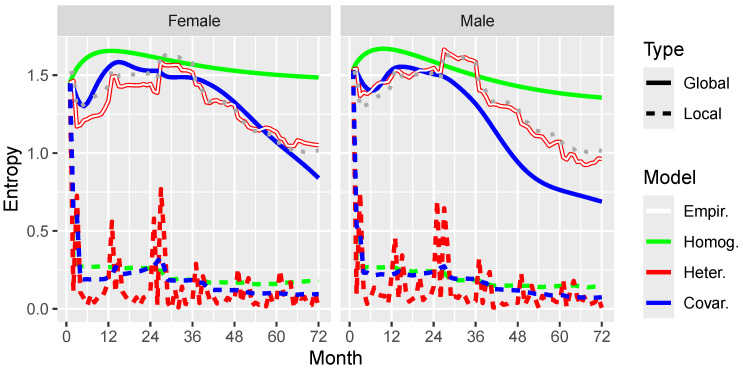
MVAD: Model-based entropy over time evaluated globally (solid lines) and locally (dashed lines) among females (**left panel**) and males (**right panel**) for three MC models: time-homogeneous, time-heterogeneous, and with individual covariates for initial and transition probabilities. Aggregated model-based entropy for the whole sample in grey dots. Cross-sectional entropy is depicted as a white solid line. The x-axis represents months after secondary education becomes non-compulsory (age 16).

**Figure A6:**
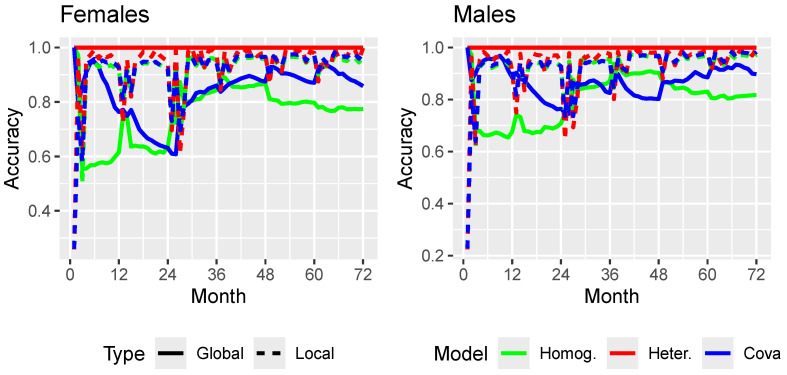
MVAD: Accuracy over time evaluated globally (solid lines) and locally (dashed lines) among females (**left panel**) and males (**right panel**) for three MC models: time-homogeneous, time-heterogeneous, and with individual covariates for initial and transition probabilities. The x-axis represents months after secondary education becomes non-compulsory (age 16).

**Figure A7:**
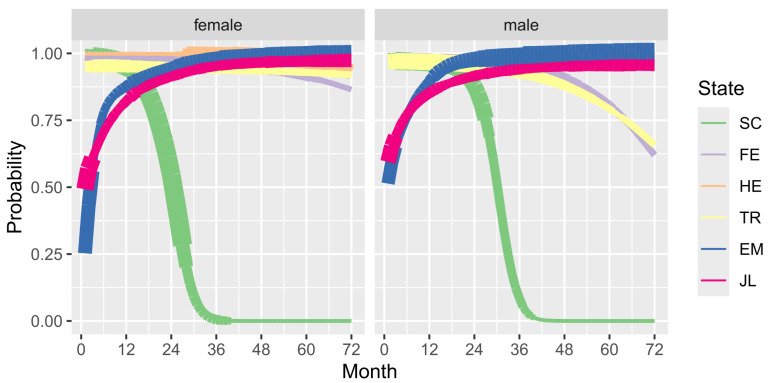
MVAD: state persistence over time with covariates for a hypothetical average individual among females (**left panel**) and males (**right panel**). The thickness of the lines captures the relative proportion of individuals in that state at that time. The x-axis represents months after secondary education becomes non-compulsory (age 16). Abbreviations: SC = school; FE = further (vocational) education; HE = higher education; TR = apprenticeship training; EM = employment; JL = jobless (not in school).

**Figure A8:**
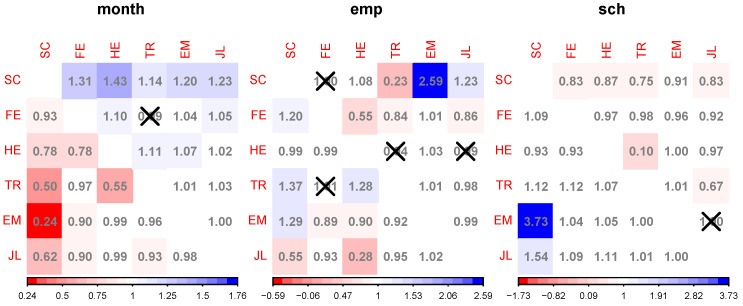
MVAD: covariate effects for females (month, emp, and sch) presented as odds ratios for the transition probabilities from one of the six states of the MC model to another state, relative to staying in the same state. The intensity of cell color (scale centered at OR = 1) indicates the relative magnitude of the estimated coefficient (within matrix). Abbreviations: SC = school; FE = further (vocational) education; HE = higher education; TR = apprenticeship training; EM = employment; JL = jobless (not in school). An X represents an n.s. coefficient.

**Figure A9:**
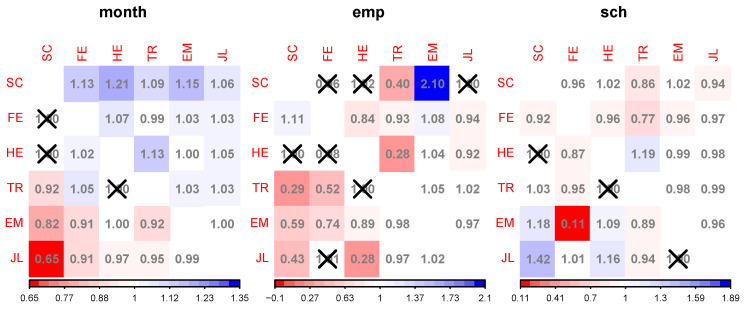
MVAD: covariate effects for males (month, emp, and sch) presented as odds ratios for the transition probabilities from one of the six states of the MC model to another state, relative to staying in the same state. The intensity of cell color (scale centered at OR = 1) indicates the relative magnitude of the estimated coefficient (within matrix). Abbreviations: SC = school; FE = further (vocational) education; HE = higher education; TR = apprenticeship training; EM = employment; JL = jobless (not in school). An X represents an n.s. coefficient.

**Figure A10:**
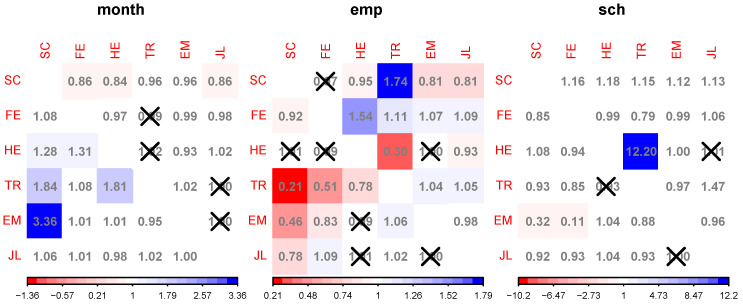
MVAD: Covariate effect for males vs. females; relative odds ratios for the transition probabilities from one of the six states of the MC model to another state, relative to staying in the same state. The intensity of cell color (scale centered at OR = 1) indicates the relative magnitude of the estimated coefficient (within matrix). Abbreviations: SC = school; FE = further (vocational) education; HE = higher education; TR = apprenticeship training; EM = employment; JL = jobless (not in school). An X represents an n.s. coefficient.

**Figure A11:**
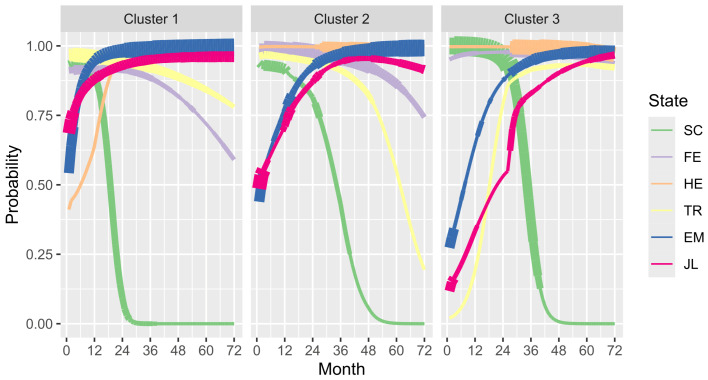
MVAD: state persistence over time with covariates for a hypothetical average individual for the three clusters solution. The thickness of the lines captures the relative proportion of individuals in that state at that time. The x-axis represents months after secondary education becomes non-compulsory (age 16). Abbreviations: SC = school; FE = further (vocational) education; HE = higher education; TR = apprenticeship training; EM = employment; JL = jobless (not in school).

## Data Availability

Data and code to replicate the analyses and figures in this article is publicly available at the following GitHub web page: https://github.com/marcascott/ModelBasedUncertainty, accessed on 8 June 2026.

## Appendix A

In this appendix we include supplemental discussion and figures referred to in the text.

### Appendix A.1. Maximum Likelihood Estimation

In the LMest package [34,35], initialization of the model parameters is generally performed in two ways. There is a deterministic approach, which consists of computing the global logits of the empirical distribution of the response variable, with the initial probabilities set equally across categories and the transition probabilities initialized using a specific rule: $\frac{1}{h+M}$ where *h* is a constant chosen to be 9; see [11] for more details on the estimation. There is a random initialization rule, which uses standardized random numbers, drawn from a uniform distribution between 0 and 1, for both the initial and transition probabilities. In the same R package, two convergence criteria are implemented. One is based on the difference in terms of log-likelihood of two consecutive steps and terminates when it is less than a suitable tolerance level. That is, considering $\theta^{\left(s\right)}$ as the parameter estimate obtained at the end of the *s*-th step of the algorithm: $l\left(\theta^{\left(s\right)}\right)-l\left(\theta^{\left(s-1\right)}\right)\leq \epsilon_{1}>0$. The other criterion is based on a minimum distance between the maximum of element-wise distance, $\max_{h}\left|\theta_{h}^{\left(s\right)}-\theta_{h}^{\left(s-1\right)}\right|\leq \epsilon_{2}>0$, where $\epsilon_{1}$ and $\epsilon_{2}$ are tolerance levels fixed for example at $10^{-8}$.

### Appendix A.2. Simulated Data

#### Appendix A.2.1. Individual-Level Entropy

Typical calculation of sequence- or individual-level entropy ignores time, pooling all observations, computing the frequency distribution and then the entropy from those probabilities. We do this for the simulated data and report an index plot for a sample of size 75 sorted by the entropy (left panel), along with the density of this measure (right panel) for the full population (black, solid) and sample (blue, dashed) and display these in Figure A1. We see that sequences 1–3 at the bottom have low entropy at the individual sequence level, given that most of their time is spent in a single state (B, B, and A, respectively). On the other hand, sequence 75 (top line) has about the same number of spells for each state, resulting in high within-sequence entropy. Note that an individual sequence may have only two changes (A to B followed by B to C) and be highly entropic if the proportion of each state in the sequence is similar (e.g. 0.33). The density for the whole population was left skewed (skew = −1.28). Rescaled to the range 0–1, it closely resembled a Beta distribution (MLEs $\hat{\alpha }=$ 4.6, $\hat{\beta }=$ 0.9 estimated using fitdistr in R MASS package [61]), which suggests higher entropy for the majority of the sequences.

#### Appendix A.2.2. Covariate Effects

Estimates of the model parameters are multinomial odds ratios, with the reference state changing to reflect the origin state; see Equation (2). To organize the parameters in a recognizable fashion, we display odds ratios in their proper location in the transition matrix, with a different matrix for each covariate in the model, all on the same scale. This matrix is for any time points t−1 to *t*, and the effects are multiplicative in the odds. The baseline matrices with probability entries can be constructed after including the constant using cascading equations familiar in multinomial logistic regression.

In Figure A2, we focus on data simulated for the illustrative example presented in Section 2.4, for which all odds ratios are greater than 1. We use shades of blue to indicate magnitude, but when odds ratios are less than one, we use red.

We see that increasing $x_{1}$ by one unit is associated with 2 or 3 times the odds of a transition from “C” to “A” or “B”, relative to remaining at “C” (bottom row). For $x_{2}$ these same odds are a bit higher. The odds of a transition leaving “B” are about twice those of remaining, and for “A” the effects are smaller, closer to 1.5 times the odds. All of these effects are significant at the 0.05 level, forming *p*-values using the estimates and their estimated standard errors provided by the lmestMc function of the LMest package relying on the standard asymptotic method, because the usual regularity conditions hold. Were something to be non-significant, we would “x” out that cell.

### Appendix A.3. Aggregated MVAD

#### Appendix A.3.1. Accuracy

Referring to the MVAD application data, Figure A3 depicts accuracy, over time, of the three models under consideration: heterogeneous MC, homogeneous MC, and covariate-dependent MC. This uses the complete MVAD dataset rather than a stratified version. Accuracy complements entropy, or rather it completes the narrative implied by entropy by verifying whether periods of lower entropy correspond to those of higher accuracy.

#### Appendix A.3.2. Covariate Effects

In Figure A4, we display the odds ratios for transitions associated with the three time-dependent covariates, month, EMP and SCH, for the model conditioned on covariates. Here, we discover which covariates have bigger impacts and on which transitions. We present the effects as relative odds, where the reference group for a transition is the self-loop. While the effects are time-constant (as odds), the covariates are time-varying, so smaller and bigger impacts may be predicted. Specific effects were highlighted in Section 6.1.5.

### Appendix A.4. Stratified MVAD

As discussed in the text, we evaluate the same set of models for data stratified by sex, a form of interaction. For the covariate model, we estimate the same model but remove sex from the initial state model, leaving only father’s professional status. The three time-varying covariates remain—we report the findings here.

#### Appendix A.4.1. Entropy

Referring to Figure A5, the average global entropy of MVAD under a model with covariates was 1.28 (grey dotted reference line), while it is 1.34 for females and 1.18 for males. We see that the differences between aggregate and stratified models are relatively small. The biggest difference is in the first few months, for which the entropy is lower for females. We know that more of them begin in school, and this could account for some reduction in uncertainty. Viewing the match of global model-based entropy to the empirical as fit, the covariate models are less well-matched for males.

#### Appendix A.4.2. Accuracy

Figure A6 depicts the accuracy of two separately estimated sets of three models using MVAD stratified by sex. There are small differences, with a bit lower accuracy for females in the first 24 months, but these separated results are quite similar to what we found when they were aggregated.

#### Appendix A.4.3. State Persistence

State persistence (analogous to autocorrelation for continuous time series) is depicted in Figure A7 separately for females (left panel) and males (right panel). As before, state persistence is calculated from the diagonal of the one-step transition matrix calculated for each month of the study, using covariate values set to the weighted mean of cumulative employment and schooling for that month, stratified by sex. For example, in month 12, females and males have accumulated 0.9 and 1 months of employment and 6.1 and 4.1 months of schooling, respectively. The differences in months of employment remain fairly minor throughout the study, with women slowly being outpaced by men for a final gap of 25.2 vs. 29.5. The gap in cumulative schooling becomes 11 vs. 7.4 by month 24, after which secondary school ends. These are the “mythical average” values associated with each month by sex.

State persistence is initially high, other than employment and joblessness, while these become increasingly persistent in the first 12 months, and the tendency appears to grow more quickly for women. This does not mean that females initiate employment or are jobless before males but rather that once they leave education their future state is more persistent over time. Notice also that training and further education states are much less persistent in the case of males as they age, when compared to females. We expect that males are leaving to initiate employment in this later period, while females seem to continue in these education and training states once they have embarked on them. Inferential questions comparing model coefficients from different groups can be made based on standard errors available in lmestMc fits.

#### Appendix A.4.4. Covariate Effects

Figure A8 and Figure A9 depict the effects of time-varying predictors for the model conditioned on covariates for MVAD stratified by sex. Contrasting these two effect summaries, we find that for both sexes, moving from schooling to all other states increases with the passage of time (month effect) but with a slightly stronger effect for females. While overall the sign is consistent with the aggregate effects given in Figure A4, we learn here that there are apparent differences for the two groups. A direct comparison is given in Figure A10, in which we take the ratio of the odds ratios, with females as reference in the denominator, which is a form of relative odds ratio (ROR). All month effects for transitions from school are significantly different across the two groups. Again, effects not significant at the 0.05 level are crossed out.

We now highlight the differential effect of cumulative employment (emp) on the transition out of schooling and higher education. For females, there is a large and positive effect (OR = 2.59) on the transition SC→EM, while it is strong, but a bit smaller for males (OR = 2.10). Their ratio (2.10/2.59; M/F) is significant and implies that (previously more-employed) males have 19% lower odds of that transition. The effect of an additional year of emp on the transition SC→TR is negative for both sexes, but their ratio suggests that (previously more-employed) males have almost twice the odds of a transition to training.

Comparing those who accumulated more prior schooling after it was non-compulsory, the effect on EM→SC transition is very large (OR = 3.73) for females but less so for males (OR = 1.18). This suggests that more prior schooling predicts a transition back to schooling from employment, and closer inspection reveals that it is concentrated in the first few months of the study. The effect of sch on the EM→FE is negligible for females (OR = 1.04) yet strongly “protective” for males (OR = 0.11). The aggregate effects were non- significant (OR = 1.02; Figure A4), while the male/female ROR is strong and significant (OR = 0.11). Closer inspection suggests that sex differences are strongest the summer period between the first and second year of non-compulsory schooling. Further examining the effect of sch, a very strong difference in the transition HE→TR for males vs. females emerges with ROR = 12.2. During the last four years of the study, once higher education is a possibility, men leave it for employment at much higher rates than women.

### Appendix A.5. Clustered MVAD

#### State Persistence

Examining model-based estimates of cluster-specific state persistence in Figure A11, we again present the diagonal of the transition matrices based on covariates for individuals specific to each cluster evolving over time. The “mythical average” individual for each cluster proceeds from the beginning of the study at 0 months of employment and post-compulsory schooling to having accumulated an average of (39.7, 23, and 12.1) months of employment and (2.9, 2.6, and 27.3) months of (secondary) school in clusters 1, 2, and 3, respectively, at the end of the study (72 months later). So, the covariates are accumulating in a different manner and these relate to the state persistence differently as well.

In cluster 1, training and schooling are quite different from each other and from the behavior in other clusters. Schooling drops off sharply, while remaining in training is highly indicated for longer than in other clusters. Further education becomes less persistent sooner than in other clusters. In cluster 2, further education has a strong persistence over time, with schooling and training dropping off sooner. Cluster 3 is the schooling cluster, dominated by persistence in school and then in higher education. Notably, initial persistence for employment and training are low, but they gain traction over time. It is important to note that the state distribution (Figure 7) and index plots (not shown) could imply some of these characterizations, such as quickly shedding further education in cluster 1. The evidence provided tends to be indirect or suggestive, because it is not model-based, nor is it always from an individual perspective. Even index plots, which depict individual trajectories, need to be organized or sorted in a manner that could reveal evidence of the type we deduce from a modeling approach.
